# Pregnancy, perinatal and childhood outcomes in women with and without polycystic ovary syndrome and metformin during pregnancy: a nationwide population-based study

**DOI:** 10.1186/s12958-022-00905-6

**Published:** 2022-02-07

**Authors:** Romina Fornes, Johanna Simin, Minh Hanh Nguyen, Gonzalo Cruz, Nicolás Crisosto, Maartje van der Schaaf, Lars Engstrand, Nele Brusselaers

**Affiliations:** 1 Centre for Translational Microbiome Research (CTMR), Department of Microbiology, Tumour and Cell Biology, Karolinska Institutet, Biomedicum kvarter 8A, Tomtebodavägen 16, SE-171 65 Stockholm, Sweden; 2 I-BioStat, Data Science Institute, Hasselt University, Hasselt, Belgium; 3 Centro de Neurobiología y Fisiopatología Integrativa (CENFI), Facultad de Ciencias, Universidad de Valparaíso, Valparaíso, Chile; 4 Laboratory of Endocrinology and Metabolism, West Division, Faculty of Medicine, University of Chile, Santiago, Chile; 5 Endocrinology Unit, Clínica Las Condes, Las Condes, Chile; 6 Department of Obstetrics and Gynaecology, Falun Hospital, Falun, Sweden; 7 Global Health Institute, Antwerp University, Antwerpen, Belgium

**Keywords:** PCOS, Metformin, Pregnancy, Obesity, Gestational, Pre-eclampsia, Diabetes

## Abstract

**Background:**

Polycystic Ovary Syndrome (PCOS) is an endocrine disorder that affects women in reproductive age and represents an unfavourable risk factor for several pregnancy and perinatal outcomes. Despite, no guidelines or pharmaceutical strategies for treating PCOS during pregnancy are available. The aim of this study is to determine the association between polycystic ovary syndrome with or without metformin and the pregnancy, perinatal outcomes as well as the risk of obesity in children born to these mothers.

**Methods:**

In this nationwide population-based cohort study based in Swedish population, all singleton births (*n* = 1,016,805) from 686,847 women since 2006 up to 2016 were included. Multivariable logistic and Cox regression modelling with odds ratios (OR) and hazard ratios (HR) and 95% confidence intervals were used to study the association between the exposure of maternal PCOS, metformin during pregnancy (or the combination of both) and: 1) Pregnancy outcomes: preeclampsia, gestational diabetes, caesarean section, and acute caesarean section, 2) Perinatal outcomes: preterm birth, stillbirth, low birth weight, macrosomia, Apgar < 7 at 5 min, small for gestational age and large for gestational age, and 3) Childhood Obesity.

**Results:**

PCOS in women without metformin use during pregnancy was associated with higher risks of preeclampsia (OR = 1.09, 1.02–1.17), gestational diabetes (OR = 1.71, 1.53–1.91) and caesarean section (OR = 1.08, 1.04–1.12), preterm birth (OR = 1.30, 1.23–1.38), low birth weight (OR = 1.29, 1.20–1.38), low Apgar scores (OR = 1.17, 1.05–1.31) and large for gestational age (OR = 1.11, 1.03–1.20). Metformin use during pregnancy (in women without PCOS) was associated with a 29% lower risks of preeclampsia (OR = 0.71, 0.51–0.97), macrosomia and large for gestational age. Obesity was more common among children born to mothers with PCOS without metformin (HR = 1.61, 1.44–1.81); and those with metformin without PCOS (HR = 1.67, 1.05–2.65). PCOS with metformin was not associated with any adverse outcome.

**Conclusion:**

PCOS was associated with increased risks of adverse pregnancy and perinatal outcomes and childhood obesity. Metformin appears to reduce these risks in mothers with polycystic ovary syndrome and their children; but may increase the risk of childhood-obesity in children form women without PCOS.

**Supplementary Information:**

The online version contains supplementary material available at 10.1186/s12958-022-00905-6.

## Introduction

Polycystic Ovary Syndrome (PCOS) is an endocrine disorder that affects approximately 10% of all women in reproductive age [1]. Despite emerging evidence suggesting that PCOS is an unfavourable risk factor for several pregnancy and perinatal outcomes, no specific guidelines or pharmaceutical treatments for during pregnancy are available, yet dietary and lifestyle modifications may include insulin sensitizer drugs [2, 3]. One of those insulin sensitizers is metformin, which reduces fasting insulin and androgen levels even in non-obese non-pregnant women [4]. Indications include Type II diabetes, obesity, metabolic syndrome, and gestational diabetes. Metformin lowers glucose-levels by the reduction of hepatic glucose production and enhancement of peripheral insulin sensitivity during pregnancy [3]. Most common side effects are diarrhoea, nausea, and stomach pain. As metformin crosses the placenta, the potential harmful effects in the developing foetus remain unclear [5]. Several studies evaluated metformin-efficacy during pregnancy and the consequences of in-utero exposure [6–21]. These suggest that women with PCOS treated with metformin may have lower risks of late miscarriage, but their children display higher body mass indexes (BMI), waist-circumferences and higher waist-to-height ratio at 5–10 years of age, compared to placebo [11, 22]. However, these studies were hampered by limited power, residual confounding by comorbidities or drug intake, and a lack of generalizability.

Therefore, in this large nationwide and population-based cohort study, we aimed to investigate whether PCOS, metformin intake or a combination of both may influence pregnancy, perinatal and childhood outcomes.

## Material and methods

This Swedish population-based cohort study included all singleton births with a recorded delivery date between July 2006 and December 2016, as identified from the Medical Birth Register.

The unique Swedish personal identity number was used to link data to 1) the National Patient Registry (in- and specialist outpatient care) which contained observations until 31st of December of 2017, 2) the Causes of Death Registry, that contains causes and dates of death (until 2017), and 3) the Swedish Prescribed Drug Registry that gathers information about drug prescriptions in the outpatient care since July 2005 until 2017. The International Classification of Disease (ICD) 8-10th edition were used to identify different diagnoses.

Ethical permission was granted by the Regional Ethics Review Board in Stockholm (2017/2423–31) without the need for informed consent.

### Exposures

The exposures were PCOS with or without metformin use during pregnancy, grouped as: i) Reference: absence of PCOS and no metformin use, PCOS(−)/metformin(−); ii) Absence of PCOS with metformin use, PCOS(−)/Metformin(+), iii) PCOS and no metformin use, PCOS(+)/metformin(−) and iv) PCOS and metformin-use, PCOS(+)/metformin(+). To evaluate the risk of preeclampsia and gestational diabetes, metformin-exposure was defined as ≥ one prescription before week 24.

PCOS was defined as having at least one recorded diagnosis (ICD-10: E282) in the Medical Birth Register during the study period or in the National Patient Registry by using the codes ICD-10: E282, ICD9: 256E or ICD8: 256,9. The ICD-code in the 8th version corresponds to Stein-Leventhal syndrome. Most of the women in Sweden are diagnosed with PCOS based on the Rotterdam criteria. Metformin use was defined as having received at least one dispensed prescription (code A10BA02 according to the Anatomical Therapeutic Chemical Classification System, ATC) during the pregnancy period, from the last menstrual period date until the delivery date. The last menstrual period date was calculated by extracting the gestational age to the birth date (as the birth date was given in year/month format due to privacy regulations, the day of birth was set as 15th in all the observations). The amount of dispensed metformin was calculated by extracting the cumulative defined daily dose (DDD) per package dispensed by the pharmacy. In 2700 women, gestational diabetes was recorded in the Medical Birth registry, while prior diagnosis of diabetes was reported in the Patient Registry. Therefore, these women were excluded from the cohort.

### Outcomes

The outcomes were grouped as 1) *pregnancy outcomes*: preeclampsia, gestational diabetes diagnosed after 24 gestational weeks (ICD10: O244), (acute) caesarean section, and 2) *perinatal outcomes*: preterm birth, stillbirth, low birth weight, macrosomia, Apgar < 7 at 5 min, small or large for gestational age (SGA or LGA), and 3) childhood-onset obesity (ICD-10: E66) before 11.5 years of age (maximal follow-up in the cohort).

The covariates extracted from the Medical Birth Register included: maternal age at delivery (stratified as < 25, 25–29, 30–34 and ≥ 35), maternal BMI (stratified as < 20, 20–24.9, 25–29.9 and ≥ 30 kg/m2), parity (nulliparous/multiparous), cigarette consumption at enrolment (yes/no), and assisted reproduction (yes/no) (Supplementary methods). Diabetes mellitus diagnosis in the patient registry during pregnancy or before (ICD10: O240, O241, E10, E11) and gestational diabetes were collected from the Medical Birth and Patient Registries. Observations of women presenting with both diagnoses, i.e., gestational diabetes and diabetes mellitus, during the same pregnancy were excluded, as well as women with missing values in any of the variables included in the model.

### Statistical analysis

Multivariable logistic regression was used to assess the risk of pregnancy and perinatal outcomes, while to determine the risk of obesity during childhood the Cox proportional Hazard modelling was used. The results were expressed as multivariable adjusted odds ratios (OR) and hazard ratios (HR) and 95% confidence intervals (CIs), respectively, using the group of women without PCOS and without metformin exposure as reference.

As no interaction between PCOS and metformin was found in any of the outcomes analysed, stratification of the participants according to the exposures was done as previously explained. The adjusted model to evaluate the association between the exposures and pregnancy and perinatal outcomes considered all covariates except in the calculation of the risk of gestational diabetes which excluded gestational diabetes and diabetes mellitus. To evaluate the association between the exposures and obesity during childhood, the model was stratified by gestational diabetes, year of birth, and adjusted by the covariates: maternal BMI, maternal age, year of birth, cigarette consumption, assisted reproduction, parity, and diabetes mellitus.

As the cohort includes women with one or multiple pregnancies, some observations may be correlated. Therefore, to evaluate perinatal outcomes, the analyses were performed using the Generalized Estimating Equation and “independence” working correlation structure that was chosen based on quasi-information criterion (QIC). In the case of the Cox proportional Hazard modelling, robust standard errors were considered in the analysis. Follow-up time was calculated from birth until the first recorded diagnosis of obesity, death, or end of study period (December 2017), whichever occurred first. The information about metformin prescription was presented as median and interquartile range (IQR).

The risk of SGA and LGA was assessed in two subgroups: i) women with diagnosis of gestational diabetes or ii) women affected by diabetes mellitus.

As the clinical charts for the diagnosis for obesity in children differs from the diagnosis in the infants (below 2 years) a sub-analysis of the risk of obesity was done excluding the children younger than 2 years.

The risk of preeclampsia and gestational diabetes at any time during pregnancy in women with metformin prescriptions was restricted to women with prescriptions in the entire pregnancy (three trimesters).

The filtering and cleaning of data was done by using PostgreSQL followed by the statistical analysis by using RStudio version 4.02 [23].

*Patient and public involvement:* there was no patient or public involvement when designing and conducting this study, but the findings will be disseminated to the public and relevant health care providers after publication.

### Funding

Becas Chile, HKH Kronprinsessan Lovisas förening för barnasjukvård and Stiftelsen Axel Tielmans minnesfond Lovisas. These funding sources were all competitive and peer-reviewed; yet were not involved in the design, collection, analysis, or interpretation of the data, writing or the decision to submit this report.

## Results

In this cohort, 1,016,805 singleton pregnancies were included, from 686,847 women. Of these, 21,833 pregnancies occurred in the 14,929 women diagnosed with PCOS (Table 1). Metformin was prescribed in 347 and 870 pregnancies with and without PCOS respectively. Of the pregnancies, 55–72% occurred in women older than 30 years, yet women with metformin (without PCOS) were mostly 35 years or older (37.0%). More than 50% of pregnant women using metformin, regardless of presence or absence of PCOS, had a BMI above 30 kg/m^2^. Women with PCOS, with and without metformin, were more likely to be primiparous (57.1 and 51.9%, respectively). Cigarette consumption at enrolment was similar across the groups while assisted reproduction was reported in approximately 5% of all pregnancies. Women using metformin were more likely to present with type I or II diabetes, irrespective of presence or absence of PCOS (14.1 and 31.4%, respectively) and gestational diabetes (7.5 and 32.4%, respectively) (Table 1).

**Table 1:** Maternal characteristics of all pregnancies in Sweden (2006—2016) categorized by presence or absence of polycystic ovary syndrome (PCOS) and use of metformin

	PCOS (−) Metformin (−) n (%)		PCOS (−) Metformin (+) n (%)		PCOS (+) Metformin (−) n (%)		PCOS (+) Metformin (+) n (%)	
	994,102		870		21,486		347	
**Maternal age**								
**< 25**	142,228	(14.3)	58	(6.7)	2875	(13.4)	34	(9.8)
**25–29**	296,675	(29.8)	185	(21.3)	7108	(33.1)	105	(30.3)
**30–34**	338,465	(34)	305	(35.1)	7547	(35.1)	122	(35.2)
**≥ 35**	216,734	(21.8)	322	(37.0)	3956	(18.4)	86	(24.8)
**Maternal BMI (Kg/m2)**								
**< 20**	103,661	(10.4)	14	(1.6)	1600	(7.4)	9	(2.6)
**20–24.9**	518,187	(52.1)	152	(17.5)	8462	(39.4)	60	(17.3)
**25–29.9**	249,843	(25.1)	234	(26.9)	6022	(28)	92	(26.5)
**≥ 30**	122,411	(12.3)	470	(54)	5402	(25.1)	186	(53.6)
**Parity**								
**Multiparous**	556,148	(55.9)	555	(63.8)	10,332	(48.1)	149	(42.9)
**Primiparous**	437,954	(44.1)	315	(36.2)	11,154	(51.9)	198	(57.1)
**Cigarette Consumption**								
**Yes**	59,804	(6)	47	(5.4)	1228	(5.7)	20	(5.8)
**No**	934,298	(94)	823	(94.6)	20,258	(94.3)	327	(94.2)
**Assisted Reproduction**								
**Yes**	28,376	(2.9)	53	(6.1)	2357	(11)	34	(9.8)
**No**	965,726	(97.1)	817	(93.9)	19,129	(89)	313	(90.2)
**Diabetes Mellitus**								
**Yes**	5695	(0.6)	273	(31.4)	304	(1.4)	49	(14.1)
**No**	988,407	(99.4)	597	(68.6)	21,182	(98.6)	298	(85.9)
**Gestational Diabetes**								
**Yes**	11,578	(1.2)	282	(32.4)	559	(2.6)	26	(7.5)
**No**	982,524	(98.8)	588	(67.6)	20,927	(97.4)	321	(92.5)

Abbreviations: *BMI* Body mass index

### Metformin intake

The median accumulated DDD for metformin among women without PCOS was similar to those with PCOS [DDD = 50 (IQR 25–100), and DDD = 55 (IQR 25–85), respectively]. When restricting to women with metformin prescriptions before 24 weeks of pregnancy, the median DDD was (DDD = 50, IQR 25–85) for women without PCOS (DDD = 50, IQR 25–75) and for women with PCOS (Table S1). For women with and without PCOS, who dispensed prescriptions during the three trimesters, the median DDD was 200 (IQR 150–276) and 175 (IQR 150–248) respectively. Respectively 57.3 and 40.2% of women with and without PCOS used metformin before the pregnancy.

### Pregnancy complications

Firstly, no interaction between PCOS and metformin was found in any of the models. Compared to women without PCOS and without metformin (reference), PCOS (without metformin) was associated with higher risk of caesarean section (OR = 1.08, 95%CI 1.04–1.12) preeclampsia (OR = 1.09, 95%CI 1.02–1.17), and gestational diabetes (OR = 1.71, 95%CI 1.53–1.91) in the fully adjusted analysis (Table 2). As the number of patients with metformin in the entire pregnancy was insufficient, the analysis considered at least one prescription of metformin before 24 weeks to evaluate the risk of preeclampsia and gestational diabetes. Metformin usage up to 24 weeks of pregnancy (regardless its use before or after), in women without PCOS, was associated with 29% decreased risk of preeclampsia, (OR = 0.71, 95%CI 0.51–0.97), when compared to reference (Table 2). Neither PCOS(−)/metformin(+) nor PCOS(+)/metformin(+) had effect in the risk of gestational diabetes (diagnosed after 24 weeks), when compared to the reference. In the sensitivity analysis including women with metformin prescriptions in all the trimesters, metformin was associated with gestational diabetes (diagnosed at any time during pregnancy) (Table S2).

**Table 2:** The risk of pregnancy complications categorized by maternal presence of polycystic ovary syndrome (PCOS) and exposure to metformin

Outcomes	PCOS(−) Metformin(−)	PCOS(−) Metformin(+)^**d**^	PCOS(+) Metformin(−)	PCOS(+) Metformin(+)^**d**^	PCOS(−) Metformin(+)^**d**^	PCOS(+) Metformin(−)	PCOS(+) Metformin(+)^**d**^	PCOS(−) Metformin(+)^d^	PCOS(+) Metformin(−)	PCOS(+) Metformin(+)^d^
	**Number (%)**				**Crude, OR (95% CI)**			**Adjusted, OR (95% CI)**^**e**^		
	**994,102**	**870**	**21,486**	**347**						
**C-Section**^**a**^										
**Yes**	164,226 (16.57)	305 (35.3)	4269 (19.96)	94 (27.09)	**2.75 (2.38–3.18)**	**1.26 (1.21–1.29)**	**1.87 (1.48–2.37)**	1.09 (0.93–1.29)	**1.08 (1.04–1.12)**	1.00 (0.77–1.30)
**No**	826,793 (83.43)	559 (64.7)	17,116 (80.04)	253 (72.91)						
**Acute C-Section**^**b**^										
**Yes**	86,627 (52.75)	155 (50.82)	2367 (55.45)	57 (60.64)	0.93 (0.74–1.16)	**1.11 (1.05–1.19)**	1.38 (0.91–2.08)	0.88 (0.69–1.13)	0.97 (0.91–1.04)	1.14 (0.75–1.74)
**No**	77,599 (47.25)	150 (49.18)	1902 (44.55)	37 (39.36)						
	**994,102**	**620**	**21,486**	**326**	**Crude, OR (95% CI)**			**Adjusted, OR (95% CI)**^**1**^		
**Preeclampsia**										
**Yes**	31,927 (3.21)	51 (8.23)	1002 (4.66)	30 (9.2)	**2.70 (2.03–3.59)**	**1.47 (1.38–1.58)**	**3.05 (2.09–4.44)**	**0.71 (0.51–0.97)**	**1.09 (1.02–1.17)**	1.13 (0.75–1.71)
**No**	962,175 (96.79)	569 (91.77)	20,484 (95.34)	296 (90.8)						
**Gestational Diabetes**^**c**^										
**Yes**	8706 (0.88)	14 (2.26)	420 (1.95)	8 (2.45)	**2.61 (1.54–4.44)**	**2.26 (2.03–2.51)**	**2.85 (1.41–5.74)**	1.15 (0.67–1.96)	**1.71 (1.53–1.91)**	1.32 (0.66–2.67)
**No**	985,396 (99.12)	606 (97.74)	21,066 (98.05)	318 (97.55)						

Abbreviations: *OR* odds ratio, *CI* confidence interval, *C-section* caesarean section

^a^ Birth alive only

^b^ Only those with C-section, Elective C-section as reference

^c^ Gestational diabetes diagnosed after 24 weeks

^d^ The risk of preeclampsia and gestational diabetes were calculated by considering just women who had at least one prescription of metformin before 24 weeks of pregnancy. Otherwise, at least one prescription during the entire pregnancy applied

^e^To calculate the adjusted risk of preeclampsia, caesarean-section and acute C-section, the variables maternal body mass index, maternal age, year of birth, cigarette consumption, assisted reproduction, parity, gestational diabetes, and diabetes mellitus were considered. To calculate the adjusted risk of gestational diabetes, the variables gestational diabetes and diabetes mellitus were excluded from the model

### Perinatal outcomes

Compared to the reference, the crude analysis shows that PCOS, metformin or PCOS combined with metformin were associated with a higher risk of developing almost all adverse outcomes studied (Table 3). However, after adjustment, PCOS(+)/metformin(−), was associated with a higher risk of preterm birth (OR = 1.30, 95%CI 1.23–1.38), stillbirth (OR = 1.30, 95%CI 1.06–1.59), as well as higher risk of low birth weight, and Apgar score < 7 at 5 min by 29 and 17%, respectively, when compared to the reference. Also comparing to the reference, PCOS (without metformin) was associated with a 11% increased risk of an LGA new-born (OR = 1.11, 95%CI 1.03–1.20) (Table 3). Metformin (without PCOS) was associated with a lower risk of macrosomia (OR = 0.75, 95%CI 0.63–0.89) and LGA (OR = 0.54, 95%CI 0.42–0.69) when compared to the reference. Overall, PCOS(+)/metformin(+) was not associated with any adverse outcome when compared to women without PCOS and no metformin (Table 3).

**Table 3:** The risk of perinatal complications categorized by maternal presence of polycystic ovary syndrome (PCOS) and exposure to metformin

	PCOS(−) Metformin(−)	PCOS(−) Metformin(+)	PCOS(+) Metformin(−)	PCOS(+) Metformin(+)	PCOS(−) Metformin(+)	PCOS(+) Metformin(−)	PCOS(+) Metformin(+)	PCOS(−) Metformin(+)	PCOS(+) Metformin(−)	PCOS(+) Metformin(+)
	Number (%)				Crude, OR (95% CI)			Adjusted, OR (95% CI)^**b**^		
	994,102	870	21,486	347						
**Preterm birth (< 37 w)**^**a**^										
**Yes**	43,597 (4.4)	91 (10.53)	1344 (6.28)	24 (6.92)	**2.56 (2.05–3.19)**	**1.46 (1.38–1.54)**	**1.62 (1.07–2.44)**	0.98 (0.78–1.24)	**1.30 (1.23–1.38)**	0.87 (0.56–1.35)
**No**	947,422 (95.6)	773 (89.47)	20,041 (93.72)	323 (93.08)						
**Stillbirth**										
**Yes**	3083 (0.31)	6 (0.69)	101 (0.47)	0 (0)	**2.23 (1.00–4.98)**	**1.52 (1.24–1.86)**	**–**	0.88 (0.39–2.02)	**1.30 (1.06–1.59)**	–
**No**	991,019 (99.69)	864 (99.31)	21,385 (99.53)	347 (100)						
**Low birth weight (< 2500 g)**										
**Yes**	30,385 (3.06)	33 (3.79)	906 (4.22)	12 (3.46)	1.25 (0.88–1.77)	**1.40 (1.30–1.50)**	1.14 (0.64–2.02)	0.92 (0.65–1.31)	**1.29 (1.20–1.38)**	0.87 (0.49–1.57)
**No**	963,717 (96.94)	837 (96.21)	20,580 (95.78)	335 (96.54)						
**Macrosomia (> 4000 g)**										
**Yes**	186,133 (18.72)	224 (25.75)	4197 (19.53)	78 (22.48)	**1.51 (1.29–1.76)**	**1.05 (1.02–1.09)**	1.26 (0.98–1.63)	**0.75 (0.63–0.89)**	0.98 (0.94–1.02)	0.86 (0.66–1.13)
**No**	807,969 (81.28)	646 (74.25)	17,289 (80.47)	269 (77.52)						
**Apgar 5 min < 7**^**a**^										
**Yes**	11,109 (1.12)	16 (1.85)	337 (1.58)	10 (2.88)	**1.66 (1.02–2.73)**	**1.41 (1.27–1.58)**	**2.62 (1.40–4.91)**	0.72 (0.44–1.2)	**1.17 (1.05–1.31)**	1.37 (0.73–2.55)
**No**	979,910 (98.88)	848 (98.15)	21,048 (98.42)	337 (97.12)						
**SGA**										
**Yes**	23,152 (2.33)	17 (1.95)	491 (2.29)	8 (2.31)	0.84 (0.52–1.35)	0.98 (0.89–1.08)	0.99 (0.49–2.00)	0.95 (0.58–1.54)	0.92 (0.84–1.01)	0.90 (0.45–1.82)
**No**	970,950 (97.67)	853 (98.05)	20,995 (97.71)	339 (97.69)						
**LGA**										
**Yes**	33,315 (3.35)	125 (14.37)	1000 (4.65)	31 (8.93)	**4.84 (3.95–5.93)**	**1.41 (1.31–1.51)**	**2.83 (1.94–4.13)**	**0.54 (0.42–0.69)**	**1.11 (1.03–1.20)**	0.75 (0.45–1.25)
**No**	960,787 (96.65)	745 (85.63)	20,486 (95.35)	316 (91.07)						

Abbreviations: *SGA* Small for Gestational Age, *LGA* Large for Gestational Age, *OR* Odds Ratio, *CI* Confidence Interval

^a^ Birth alive only

^b^ Adjusted by maternal body mass index, maternal age, year of birth, cigarette consumption, assisted reproduction, parity, gestational diabetes, and diabetes mellitus

Table S3 shows the subgroup analysis of women with gestational diabetes and diabetes mellitus. In women with gestational diabetes, PCOS, with metformin use, was related to a higher risk of LGA (OR = 2.98, 95% CI 1.26–7.08). In women with diabetes mellitus, PCOS was associated to a 29% decreased risk of delivering a child LGA, whereas metformin, in women without and with PCOS, the risk of having a baby LGA decreased by 27 and 68% respectively when compared to the reference.

### Obesity during childhood

The median follow-up of the children was 5.8 years (IQR 3.47–8.47). In the fully adjusted model, being born to a mother with PCOS without metformin use, was associated with an increased risk of developing infant obesity (HR = 1.61, 95%CI 1.44–1.81, Table 4) compared to children born to mother without PCOS and not exposed to metformin. Metformin in women without PCOS showed an association with obesity in childhood (HR = 1.67, 95%CI 1.05–2.65, Table 4). Lastly, PCOS(+)/Metformin(+) had no effect in obesity during childhood. The sub-analysis excluding the children younger than 2 years did not change the estimates substantially (Table S4).

**Table 4:** Obesity during childhood categorized by maternal presence of polycystic ovary syndrome (PCOS) and exposure to metformin

	PCOS(−) Metformin(−)	PCOS(−) Metformin(+)	PCOS(+) Metformin(−)	PCOS(+) Metformin(+)	PCOS(−) Metformin(+)	PCOS(+) Metformin(−)	PCOS(+) Metformin(+)	PCOS(−) Metformin(+)	PCOS(+) Metformin(−)	PCOS(+) Metformin(+)
	**Number (%)**				**Crude, HR (95% CI)**			**Adjusted, HR (95% CI)**^**a**^		
	**991,019**	**864**	**21,385**	**347**						
**Obesity**										
**Yes**	7589 (0.77)	19 (2.2)	319 (1.49)	6 (1.73)	**4.03 (2.57–6.31)**	**2.37 (2.11–2.66)**	**2.48 (1.12–5.49)**	**1.67 (1.05–2.65)**	**1.61 (1.44–1.81)**	1.13 (0.51–2.53)
**No**	983,430 (99.23)	845 (97.8)	21,066 (98.51)	341 (98.27)						

Abbreviations: *HR* Hazard Ratio, *CI* Confidence Interval

^a^ Adjusted by maternal body mass index, maternal age, year of birth, cigarette consumption, assisted reproduction, parity, gestational diabetes, and diabetes mellitus

## Discussion

### Main findings

The analysis of this large population-based cohort shows that PCOS was associated with an increased risk of adverse pregnancy and perinatal outcomes, as well as obesity in their children. Despite that metformin seems to be safe during pregnancy in women with PCOS, it is important to highlight that in women without PCOS, metformin intake during pregnancy was associated with an increased risk of obesity during childhood. However, we cannot distinguish between the effect of the drug and the underlying indication. Whilst promising, the results need to be interpreted with caution before clinical implementation, since more research is needed to clarify the effect of PCOS in the offspring health, but also the long-term effects of metformin and other drugs used during pregnancy, beyond teratogenic consequences.

### Strengths and limitations

To our knowledge this is the largest population-based cohort study evaluating pregnancy and perinatal outcomes in women with and without PCOS, and metformin use. The use of the registries enhances the generalizability of the results to countries with similar economic development, access to health care, treatment praxis and lifestyle. Socio-economic variables were not collected, yet access to healthcare is equal for all Swedish residents, and especially ante- and perinatal care is heavily subsidized and standardized. The group of women with PCOS receiving metformin remains small (*N* = 347) which does reflect clinical practice during the study period. Moreover, the present study shows that other conditions related to PCOS, including diabetes mellitus and gestational diabetes might play a role in the efficacy of metformin and its use in pregnancy. Therefore, the efficacy of metformin may depend on population characteristics, including incidence and intensity of insulin resistance, genetic and environmental factors. For instance, our previous studies showed that the risk for developing gestational diabetes in Chilean women with PCOS treated with metformin was 3.4-fold compared to a group of Argentinian women (also showing a higher risk of pregnancy hypertension) [24]. Unfortunately, in this study the amount of pregnancies with metformin exposure was not sufficient for doing a sub-analysis by ethnic origin.

A general concern is the validity of our diagnostics codes. The prescribed Drug Registry is virtually complete and metformin is not available over the counter [25]. Although we do not have information on actual drug-intake, women with more prescriptions (and a larger accumulated dosage) are expected to be more compliant. By including women with only one metformin prescription, this may have resulted in a misclassification of exposure if women stopped prematurely because of side-effects – most likely leading to a dilution or underestimation of the effects.

The validity of the Swedish Patient and Birth Registries is generally high; yet there may be issues for specific diagnoses, including PCOS, pre-eclampsia and obesity. Only 2.2% of the included women had recorded PCOS diagnoses, while 10–18% were expected in the general population (disregarding the established subfertility) leading to a differential misclassification and potential dilution of the effects [26–29]. Underdiagnosing and underreporting PCOS remains an important clinical issue in most settings where PCOS can only be diagnosed in case of clinical symptoms including subfertility, with the more severe cases being most likely to be detected. The ICD-codes for PCOS were insufficiently detailed to evaluate different phenotypes. Childhood obesity is also underreported, with an up to 18% expected prevalence of overweight/obesity in children between 6 and 9 years [30], compared to less than 1% in our study. Another limitation is the potential misclassification of gestational diabetes and diabetes. Regarding the risk of gestational diabetes, we only examined exposure during the early pregnancy (24 weeks), to avoid reverse causality. Moreover, in Sweden the diagnostic criteria of gestational diabetes vary across the country [31, 32]. Power was also too low to assess the risk in women exposed to metformin during all trimesters of the pregnancy. Another consideration is that women receiving metformin because of other indications rather than PCOS (obesity, metabolic syndrome, or insulin resistance) are more prone to develop gestational diabetes [33], so-called confounding by indication. Lastly, despite available insulin prescriptions, we opted not to adjust for this, but to adjust for gestational diabetes and diabetes mellitus to include more observations and avoid collinearity in our analysis.

### Interpretation

Our results confirm previous studies showing that PCOS is associated with gestational diabetes [26, 34–39], and an increased risk of preeclampsia [26, 37], preterm birth [26, 35, 40–42] and caesarean section [35, 43–45]. Although a higher risk for preterm birth and low birth weight in women with PCOS was found, the risk of acute C-section was not increased, yet preeclampsia seemed more common [46]. However, the risk for delivering a baby small for gestational age was not increased, suggesting that the low birth weight is related to preterm birth and not because an intrauterine growth restriction.

We found an association between PCOS and stillbirth, which was not reported in a previous Swedish cohort (1995–2007) [26]. As in our cohort the percentage of stillbirths from women with PCOS is 7-fold higher than the percentage reported in their cohort, the power of the previous study may have been too limited. Yet, stillbirths remain rare, and residual confounding may still play a role.

Higher BMI, gestational diabetes, diabetes mellitus and PCOS are related to a higher risk of LGA [14, 37]. The effect of PCOS+metformin or PCOS without metformin (although with a marginal non-significance) on the risk of LGA was seen when restricting to women with gestational diabetes, but not in women with diabetes mellitus. In fact, in women with diabetes mellitus, the risk of LGA was decreased in women with or without PCOS, regardless of the use of metformin. This could potentially be due to earlier and prolonged dietary or pharmacological treatment before or during pregnancy [47].

Our large database and the possibility of linking our data with the drug register, allowed us to deepen the analysis and offers a step forward into the assessment of the efficacy and safety of pharmaceutical treatments. In our analysis, metformin during pregnancy, regardless of presence or absence of PCOS, was not associated with an increased risk of caesarean section, preterm birth, lower birth weight and low Apgar scores. Therefore, metformin could be useful in the antenatal management of women with PCOS or other disorders with altered glucose metabolism, as shown before [6–8, 10, 19, 48, 49]. On the other hand, studies of women with gestational diabetes, diabetes mellitus or obesity show that the risk of preeclampsia, caesarean section or preterm birth is lower [14, 15] or similar when the effect of metformin is compared to the placebo or to insulin [15–17, 20]. In fact, in our analysis the risk of preeclampsia was lower in women without PCOS with metformin (at least before 24 weeks). Metformin-use before 24 weeks was not associated with an increased risk of gestational diabetes in women with or without PCOS, which could be influenced by potential underreporting, confounding by indication and reverse causality. We acknowledge that the ideal analysis would have considered the use of metformin during the entire pregnancy, but our sensitivity analysis considering the women with at least one prescription in each of the three trimesters lacked power. Previous studies have shown no better results regarding the ability of metformin to prevent gestational diabetes. Neither studies in PCOS, [11] nor studies in women with obesity or insulin resistance have found a clear decreased risk of gestational diabetes [50, 51].

To our knowledge, this is the first study that has evaluated LGA and SGA accounting for other comorbidities associated with PCOS. PCOS was associated with higher risk of LGA in patients with gestational diabetes and the risk was even higher in women with PCOS exposed to metformin. Previously, metformin was associated with an increased risk of higher birth weight at delivery when compared to insulin in women with a poor glycaemic control, but not in those women with a good glucose metabolism [52]. This suggests that women with PCOS treated with metformin could have had a poorer glycaemic control or that PCOS and metformin had a synergic effect in this outcome. On the other hand, in women with diabetes mellitus, both, PCOS(−)/metformin(+) and PCOS+/metformin(+), were associated with a lower risk of LGA. This “shift” towards a decreased birth weight in patients with diabetes mellitus was also reported in women with type II diabetes randomized to receive metformin or placebo on top of insulin [47]. Of note, SGA was not often annotated in our cohort, which is similar to a previous study [26].

Interestingly, we found an association between maternal metformin consumption in women without PCOS and obesity during childhood, but not in women with PCOS, as it was previously shown in studies in women with PCOS or gestational diabetes [11, 22, 52]. Despite likely underreporting of childhood obesity, the power should have been sufficient. In small follow-up studies (25 and 141 children) in mothers with PCOS exposed to metformin or placebo, metformin showed no effect on children anthropometry [12] or an increased BMI and adiposity measurements, except for mothers with high BMI.^2^ [11] In mothers with gestational diabetes, the BMI of 9-year old children exposed to antenatal metformin was only higher when mothers had a higher pregnancy-BMI (compared to insulin) [52, 53]. In our study we adjusted for maternal BMI, yet power was too limited for stratified analyses.

Metformin has shown to decrease androgen levels in PCOS women [54, 55], but this is not clear yet during pregnancy [49, 56]. Metformin improves the overall insulin sensitivity and inhibits hepatic glucose production through increasing AMP/ATP ratio and activating AMP-activated protein kinase (AMPK) [57]. In addition, metformin suppresses glucagon signalling through decreasing cAMP production in the liver and regulates nutrient sensing pathways within the placenta [58, 59]. Other mechanisms could be also involved including the regulation of the sympathetic nerve activity and angiogenesis, that are in fact, associated to diabetes, obesity, PCOS, preeclampsia and metabolic syndrome [59–61]. Also, metformin has shown to decrease antimüllerian hormone (AMH) levels in PCOS women [62, 63]. Since AMH levels remain abnormally high during PCOS pregnancy [64] and is related to transgenerational PCOS transmission [65, 66], a possible reduction in AMH levels due metformin consumption during pregnancy would account for some beneficial effects of the drug. Lastly, we should recognize the ability of metformin of modifying the composition and the metabolic function of the gastrointestinal microbiome as well as its interaction with the host [67]. This interaction is fundamental for the activation of the gut-brain-liver feedback system in the regulation of the nutrient sensing and hepatic glucose production [57]. Women with PCOS as well as women with obesity or diabetes have altered microbiome compostions [68]. As the baby is colonized with the microbiome from the mother, a “healthier” microbiome transferred to the offspring in the group of PCOS+metformin could partially explain the lack of association with obesity.

## Conclusion

In conclusion, our findings confirm that maternal PCOS may increase the risk of several adverse pregnancy and obesity in children, whereas exposure to metformin may lower this risk. Metformin in women without PCOS was associated with an increased risk of obesity in children yet confounding by indication cannot be ruled out.

## Supplementary Information

**Additional file 1.**

## Data Availability

The original data for this study belong to the National Board of Health and Welfare (Socialstyrelsen) and provided to NB after an approved application by the Regional Ethics Committee of Stockholm. The data are stored in coded format in a server at Karolinska Institutet. As the data gathered for this study are protected by Swedish law, they are not to be publicly available. However, data could be available upon reasonable request to the author responsible for the data (NB) after a relevant ethical and data-sharing approval obtained that should be in line with the General Data Protection Regulation. The underlying analysis plan is available from the authors upon request.

## Abbreviations

AMH: Antimüllerian hormone
AMPK: AMP-activated protein kinase
BMI: Body mass index
CI: Confidence interval
OR: Odds ratio
DDD: Defined daily dose
FDA: Food and Drug Administration
HR: Hazard ratios
ICD: International Classification of Disease
IQR: Interquartile range
PCOS: Polycystic Ovary Syndrome
