# Supplementary material for: Pregnancy, perinatal and childhood outcomes in women with and without polycystic ovary syndrome and metformin during pregnancy: a nationwide population-based study
Source: Reprod Biol Endocrinol. 2022 Feb 7;20:30. doi: 10.1186/s12958-022-00905-6 (PMC8819934; doi:10.1186/s12958-022-00905-6)
Supplement: Supplementary file 1 — Additional file 1. [file 12958_2022_905_MOESM1_ESM.docx]

**Supporting information**

**Supplementary methods**

**Supplementary table 1 (Table S1). Metformin prescribed and dispensed by the pharmacy during pregnancy.**

**Supplementary table 2 (Table S2). The risk of preeclampsia and gestational diabetes categorized by maternal presence of polycystic ovary syndrome (PCOS) and exposure to metformin in each trimester.**

**Supplementary table 3 (Table S3). Small or large for gestational age. Stratification by gestational diabetes or diabetes mellitus.**

**Supplementary table 4 (Table S4). Obesity in children older than 2 years of age, categorized by maternal presence of polycystic ovary syndrome (PCOS) and exposure to metformin.**

**Supplementary methods – Definition and sources of variables included in the study.**

| **Disease/Observation** | **Comments** | **International Classification of Diseases coding** | **Medical birth register** | **National Patient Registry** | **Prescribed Drug register** |
| --- | --- | --- | --- | --- | --- |
| **Maternal and pregnancy characteristics** |  |  |  |  |  |
| Smoking | Reported at registration in the prenatal care |  | yes |  |  |
| Maternal body mass index | Calculated as Kg/m2 from Height and maternal body weight reported at registration in the prenatal care |  | yes |  |  |
| Parity | Multiparous/primiparous |  | yes |  |  |
| Assisted Reproduction |  |  | yes |  |  |
| PCOS | ICD8: Stein-Leventhal’s syndrome. ICD-9 and ICD-10 diagnose specified as polycystic ovary syndrome. | ICD10:E28.2; ICD9: 256E; ICD8: 256.90 | yes | yes |  |
| Preeclampsia | Blood pressure ≥ 140/90 mmHg accompanied by proteinuria of more than 0.3 g /24 h or ≥1 on a urine dipstick | ICD10: O14 | yes |  |  |
| Gestational Diabetes | Definition varied across time (most accepted definition is the definition by WHO,2013) (1) | ICD10: O244 | yes | yes |  |
| Diabetes mellitus type 1 or 2 | National patient registry (before or during pregnancy) | ICD10: O240, O241, E10, E11 | yes | yes |  |
| Caesarean-section |  |  | yes |  |  |
| Acute caesarean section |  |  | yes |  |  |
| **Medication** |  |  |  |  |  |
| Metformin | ATC-code A10BA02 |  |  |  | yes |
| Insulin | ATC-code A10A |  |  |  | yes |
| **Perinatal and infant outcomes** |  |  |  |  |  |
| Small for gestational age | Birth weight below the 10^th^ percentile (2) |  | yes |  |  |
| Large for gestational age | Birth weight above the 90^th^ percentile (2) |  | yes |  |  |
| Stillbirth | Death before or during delivery |  | yes |  |  |
| Neonatal death | Death between 0-27 days of life |  | yes |  |  |
| Apgar <7 at 5 min | Categorized from score reported in MBR |  | yes |  |  |
| Low birth weight | Calculated from birth weight (< 2500g) |  | yes |  |  |
| Macrosomia | Calculated from birth weight (> 4000g) |  | yes |  |  |
| Preterm birth | Categorized as birth below 37 weeks of pregnancy |  | yes |  |  |
| Obesity/overweight in children |  | ICD10: E66 |  | yes |  |

Abbreviations: MBR: Medical birth register; NPR: National patient register; ICD: International classification of diseases.

1. Fadl, H., et al., *Changing diagnostic criteria for gestational diabetes in Sweden - a stepped wedge national cluster randomised controlled trial - the CDC4G study protocol.* BMC Pregnancy and Childbirth, 2019. **19**(1): p. 398.

2.Marsál, K., et al., *Intrauterine growth curves based on ultrasonically estimated foetal weights.* Acta Paediatr, 1996. **85**(7): p. 843-8.

Supplementary information 2. Exclusion criteria.

**Supplementary methods: Flow chart and exclusion criteria.**

**MediMaMi cohort**

**Medical birth Register**

Births in Sweden (July 2006 to December 2016)

(1,132,832)

- - Twins (32,352)
  - Last menstrual period not available (624)
  - Children with incomplete mother´s register (264)
  - Maternal BMI not available (74,538)
  - Apgar score not available (alive births) (4,222)
  - SGA, AGA or LGA not available (1269)
  - Inconsistencies in twin’s classification-parity (8)
  - Inconsistencies between diagnoses diabetes mellitus and gestational diabetes (by ICD code) (2750)

**Final cohort**

(1,016,805)

**Drug Register**

(2005–2017)

**Death Register**

(2005–2017)

**National Patient Register**

In-patient

Out-patient

**Table S1. Metformin prescribed and dispensed by the pharmacy during pregnancy.**

|  | **PCOS (-)  Metformin (+)** | |  | **PCOS (+)  Metformin (+)** | |
| --- | --- | --- | --- | --- | --- |
| **Metformin (period intake)** | Number | DDD Median (IQR) |  | Number | DDD Median (IQR) |
| **Metformin during pregnancy** | 870 | 50 (25-100) |  | 347 | 50 (25-85) |
| **Metformin up to 24 weeks** | 620 | 50 (25-85) |  | 326 | 50 (25-75) |
| **Metformin third trimester** | 46 | 175 (150-248) |  | 10 | 200 (150-276) |

Abbreviations: PCOS: Polycystic ovary syndrome; IQR: Interquartile range; DDD: Defined daily dose

**Table S2. The risk of preeclampsia and gestational diabetes categorized by maternal presence of polycystic ovary syndrome (PCOS) and exposure to metformin in all trimester.**

|  |  | **PCOS(-) Metformin(-)** | **PCOS(-) Metformin(+)^#^** | **PCOS(+) Metformin(+)^#^** | **PCOS(-) Metformin(+)^#^** | | **PCOS(-) Metformin(+)**^#^ | |  |
| --- | --- | --- | --- | --- | --- | --- | --- | --- | --- |
|  |  | **Number (%)** | | | **Crude, OR (95% CI)** | | **Adjusted, OR (95% CI)^1^** | | |
|  | | **994,376** | **46** | **10** |  |  |  |  |  |
| **Preeclampsia** | |  |  |  |  |  |  |  |  |
|  | **Yes** | 31,927 (3.21) | 4 (8.7) | 2 (20.00) | **-** | | **-** | |  |
|  | **No** | 962,175 (96.79) | 42 (91.3) | 8 (80.00) |  |  |  |  |  |
| **Gestational Diabetes*** | |  |  |  |  |  |  |  |  |
|  | **Yes** | 11,578 (1.16) | 5 (10.87) | 0 (0) | 10.35 (4.07-26.32) | | **3.89 (1.42-10.67)** | |  |
|  | **No** | 982,524 (98.84) | 41 (89.13) | 10 (100) |  |  |  |  |  |

Abbreviations: OR: odds ratio, CI: confidence interval, C-section: caesarean section.

* gestational diabetes diagnosed at any time during pregnancy.

# The risk of preeclampsia and gestational diabetes were calculated by considering just women who had at least one prescription of metformin in each trimester.

Power was insufficient for the subgroup analysis on PCOS(+)/Metformin(+)

^1^ To calculate the adjusted risk of preeclampsia, caesarean-section and acute C-section, the variables maternal body mass index, maternal age, year of birth, cigarette consumption, assisted reproduction, parity, gestational diabetes, and diabetes mellitus were considered. To calculate the adjusted risk of gestational diabetes, the variables gestational diabetes and diabetes mellitus were excluded from the model.

**Table S3. Small or large for gestational age. Stratification by gestational diabetes or diabetes mellitus.**

|  | |  | **PCOS (-) Metformin (-)** | **PCOS (-) Metformin (+)** | **PCOS (+) Metformin (-)** | **PCOS (+) Metformin (+)** | **PCOS (-) Metformin (+)** | **PCOS (+) Metformin (-)** | **PCOS (+) Metformin (+)** | **PCOS (-) Metformin (+)** | **PCOS (+) Metformin (-)** | **PCOS (+) Metformin (+)** |
| --- | --- | --- | --- | --- | --- | --- | --- | --- | --- | --- | --- | --- |
|  |  |  | **Number (%)** | | | | **Crude, OR (95% CI)** | | | **Adjusted, OR (95% CI)^1^** | | |
| **Gestational Diabetes** | | | **11,578** | **282** | **559** | **26** |  |  |  |  |  |  |
|  | **Large for gestational age** | | | | | | | | | | | |
|  |  | **Yes** | 1,478 (12.77) | 34 (12.06) | 96 (17.17) | 9 (34.62) | 0.94 (0.64-1.38) | **1.42 (1.12-1.79)** | **3.62 (1.58-8.26)** | 0.67 (0.44-1.01) | 1.25 (0.98-1.58) | **2.98 (1.26-7.08)** |
|  |  | **No** | 10,100 (87.23) | 248 (87.94) | 463 (82.83) | 17 (65.38) |  |  |  |  |  |  |
| **Small for gestational age** | | | | | | | | | | | | |
|  |  | **Yes** | 247 (2.13) | 2 (0.71) | 12 (2.15) | 1 (3.85) | - | 1.01 (0.56-1.81) | **-** | **-** | 1.01 (0.56-1.83) | **-** |
|  |  | **No** | 11,331 (97.87) | 280 (99.29) | 547 (97.85) | 25 (96.15) |  |  |  |  |  |  |
| **Diabetes Mellitus** | | | **5,695** | **273** | **304** | **49** |  |  |  |  |  |  |
| **Large for gestational age** | | | | | | | | | | | | |
|  |  | **Yes** | 1,934 (33.96) | 72 (26.37) | 80 (26.32) | 7 (14.29) | **0.69 (0.52-0.93)** | **0.69 (0.52-0.94)** | **0.32 (0.13-0.79)** | **0.63 (0.47-0.85)** | **0.69 (0.50-0.94)** | **0.32 (0.13-0.76)** |
|  |  | **No** | 3,761 (66.04) | 201 (73.63) | 224 (73.68) | 42 (85.71) |  |  |  |  |  |  |
| **Small for gestational age** | | | | | | | | | | | | |
|  |  | **Yes** | 93 (1.63) | 8 (2.93) | 8 (2.63) | 0 (0) | 1.82 (0.87-3.79) | 1.63 (0.78-3.41) | **-** | 1.51 (0.66-3.47) | 1.31 (0.61-2.83) | **-** |
|  |  | **No** | 5,602 (98.37) | 265 (97.07) | 296 (97.37) | 49 (100) |  |  |  |  |  |  |

Abbreviations: OR: Odds Ratio; CI: Confidence Interval.

^1^ adjusted by maternal body mass index, maternal age, year of birth, cigarette consumption, assisted reproduction and parity.

**Table S4. Obesity in children older than 2 years of age, categorized by maternal presence of polycystic ovary syndrome (PCOS) and exposure to metformin.**

|  |  | **PCOS(-) Metformin(-)** | **PCOS(-) Metformin(+)** | **PCOS(+) Metformin(-)** | **PCOS(+) Metformin(+)** | **PCOS(-) Metformin(+)** | **PCOS(+) Metformin(-)** | **PCOS(+) Metformin(+)** | **PCOS(-) Metformin(+)** | **PCOS(+) Metformin(-)** | **PCOS(+) Metformin(+)** |
| --- | --- | --- | --- | --- | --- | --- | --- | --- | --- | --- | --- |
|  |  | **Number (%)** | | | | **Crude, HR (95% CI)** | | | **Adjusted, HR (95% CI)^1^** | | |
| **All** |  | **887,243** | **616** | **18,511** | **289** |  |  |  |  |  |  |
| **Obesity** | |  |  |  |  |  |  |  |  |  |  |
|  | **Yes** | 7,395 (0.83) | 18 (2.92) | 309 (1.67) | 6 (2.08) | **3.94 (2.48-6.26)** | **2.37 (2.11-2.66)** | **2.55 (1.15-5.65)** | **1.62 (1.00-2.61)** | **1.61 (1.43-1.81)** | 1.16 (0.52-2.58) |
|  | **No** | 879,848 (99.17) | 598 (97.08) | 18,202 (98.33) | 283 (97.92) |  |  |  |  |  |  |

Abbreviations: HR: Hazard Ratio; CI: Confidence Interval.

^1^ Adjusted by maternal body mass index, maternal age, year of birth, cigarette consumption, assisted reproduction, parity, gestational diabetes, and diabetes mellitus.
